# Immune dysfunction drives adverse mpox outcomes among people living with human immunodeficiency virus: a systematic review and meta-analysis with CD4-based risk stratification

**DOI:** 10.3389/fmed.2026.1895518

**Published:** 2026-07-29

**Authors:** Chunting Qiu, Defa Zhang, Ziyu Wang, Lina Fan, Ping Ma

**Affiliations:** 1 Department of Infectious Diseases, The Second People’s Hospital of Tianjin Medical University, Tianjin, China; 2 State Key Laboratory for Diagnosis and Treatment of Severe Zoonotic Infectious Diseases, Wuhan, China

**Keywords:** CD4 count, hospitalization, human immunodeficiency virus, immunosuppression, mpox

## Abstract

**Background:**

People living with human immunodeficiency virus (HIV) accounted for a large share of cases during the 2022 multinational mpox outbreak, but whether adverse outcomes are attributable to HIV infection itself or to the degree of immune dysfunction remains uncertain.

**Methods:**

We conducted a systematic review and meta-analysis according to PRISMA 2020 and Cochrane methods. PubMed, Embase, and Web of Science were searched from inception through January 31, 2026, with targeted literature updating through May 27, 2026. Observational studies reporting mpox outcomes stratified by HIV status were eligible. The primary outcome was hospitalization. Risk ratios (RRs) were pooled using DerSimonian-Laird random-effects models.

**Results:**

Twenty studies were included in the qualitative synthesis and 13 studies comprising 32,685 participants were included in the hospitalization meta-analysis. HIV infection was associated with increased hospitalization risk (RR, 1.40; 95% CI, 1.07–1.85; I^2^ = 81%). The 95% prediction interval was wide (0.54–3.65), indicating that context, case mix, and hospitalization thresholds influenced observed effects. CD4-stratified evidence was not sufficiently uniform for a valid pooled CD4-specific effect estimate, but available studies consistently indicated that low CD4 count, uncontrolled HIV viremia, and disengagement from HIV care identify the subgroup at greatest risk for hospitalization, severe disease, prolonged infection, and death.

**Conclusion:**

Adverse mpox outcomes among people living with HIV are better explained by immune status and HIV disease control than by HIV serostatus alone. CD4-based risk stratification provides a clinically actionable framework for triage, follow-up intensity, antiviral-treatment consideration, and integration of mpox management with HIV care.

## Introduction

Mpox re-emerged as a major global public health problem during the 2022 multinational outbreak, which demonstrated sustained human-to-human transmission across multiple regions and sexual networks (1–3). Although many infections are self-limited, clinically significant disease can occur, including severe mucocutaneous involvement, systemic complications, need for hospitalization, and death in selected high-risk groups (2–4). Identifying determinants of adverse outcomes is therefore important for clinical triage, treatment prioritization, and outbreak response.

People living with HIV were disproportionately represented among mpox cases in several cohorts and surveillance datasets (3, 4). Early reports raised concern that HIV infection might be associated with more severe mpox, including higher hospitalization rates and prolonged disease (3, 4). Subsequent studies produced heterogeneous findings: some cohorts found increased hospitalization among people living with HIV, whereas others showed similar clinical courses when HIV was virologically controlled and CD4 counts were preserved (5–7).

A central limitation of the literature is that HIV infection has often been treated as a binary exposure. HIV disease spans a broad spectrum from durable viral suppression with preserved CD4 T lymphocyte counts to advanced immunosuppression with uncontrolled viremia. Emerging data indicate that immune status, rather than HIV serostatus alone, is the biologically plausible determinant of severe mpox (4–7). In a global case series of advanced HIV infection, severe necrotizing and disseminated mpox, multiorgan involvement, and all reported deaths occurred among individuals with low CD4 counts, particularly below 200 cells/mm^3^ (4).

Prior systematic reviews have suggested that HIV infection or immunosuppression may be associated with adverse mpox outcomes, but many analyses were limited by binary HIV classification, heterogeneity in outcome definitions, and limited incorporation of CD4-based risk stratification (8–10). A systematic review further confirmed adverse associations for HIV coinfection but did not fully resolve whether the signal was driven by immune dysfunction or HIV serostatus as a standalone marker (10). We therefore conducted a systematic review and meta-analysis to estimate hospitalization risk in mpox by HIV status and to synthesize evidence on immune status, particularly CD4 count and HIV disease control, as effect modifiers.

## Methods

### Study design and reporting standards

This systematic review and meta-analysis evaluated the association between HIV infection, immune status, and clinical outcomes among patients with mpox. The review was conducted according to the Cochrane Handbook for Systematic Reviews of Interventions, version 6.5, and reported according to the PRISMA 2020 statement (11, 12). A structured protocol was developed before extraction to define the research question, eligibility criteria, outcomes, and analytic plan. The protocol was not registered. Study selection, data extraction, and risk-of-bias assessment were performed independently by two reviewers, with disagreements resolved by consensus.

### PICOS framework

The population comprised human participants with mpox. The exposure was HIV infection and, where available, immune status among people living with HIV. The comparator was mpox in individuals without HIV or, for immune-status analyses, people living with HIV with preserved immune function. Outcomes were hospitalization, severe disease, prolonged disease, and mortality. Eligible study designs were observational cohort, case-control, cross-sectional, and surveillance analyses with extractable HIV-stratified outcomes.

### Data sources and search strategy

PubMed, Embase, and Web of Science were searched from database inception through January 31, 2026. Search terms combined mpox-related terms with HIV-related terms and outcome-related terms. Search strings were adapted to each database and are provided in Supplementary Appendix 1. No language or country restrictions were applied at the search stage. Reference lists of included studies and relevant reviews were screened manually. A targeted pre-submission literature check through May 27, 2026, identified major post-search publications relevant to interpretation; these studies were cited narratively when they did not alter the prespecified quantitative dataset (10, 13).

### Eligibility criteria

Eligible studies were observational human studies that included patients with confirmed or clinically adjudicated mpox and reported clinical outcomes stratified by HIV status. Studies were required to provide extractable event counts, proportions, or study-level effect estimates for at least one prespecified outcome. Case reports, case series with fewer than 10 participants, narrative reviews, editorials, commentaries, conference abstracts without sufficient data, and laboratory-only studies without clinical endpoints were excluded. When publications described overlapping populations, the report with the most complete outcome data, largest sample size, or most mature dataset was retained for the primary analysis.

### Study selection and data extraction

Two reviewers independently screened titles and abstracts, reviewed potentially eligible full texts, and extracted study-level data using a standardized form. Extracted variables included first author, publication year, country or region, study design, sample size, HIV status, hospitalization events, CD4 count, HIV RNA, antiretroviral therapy status, engagement in HIV care, and available severity or mortality outcomes. Missing data were not imputed.

### Outcomes

The primary outcome was hospitalization associated with mpox, as defined by the original investigators. Hospitalization was selected because it was the most consistently reported clinically interpretable endpoint across studies. Secondary outcomes were severe disease and mortality. Severe disease was accepted according to study-specific criteria, including extensive or necrotic skin disease, mucosal disease, systemic complications, organ involvement, need for advanced medical support, or investigator-defined severe presentation. Prespecified effect modifiers were CD4 count, HIV RNA suppression, antiretroviral therapy status, and engagement in HIV care.

### Risk-of-bias assessment

Methodological quality was assessed with the Newcastle-Ottawa Scale, adapted as appropriate for observational designs (14). The scale evaluates selection, comparability, and outcome domains, with a maximum score of 9 points. Studies scoring 7 or higher were considered high quality, scores of 5–6 moderate quality, and scores below 5 low quality. Particular attention was paid to confounding by age, comorbidities, case ascertainment, healthcare access, hospitalization threshold, and immune status.

### Statistical analysis

For dichotomous outcomes, study-level RRs and 95% confidence intervals (CIs) were calculated from extracted event counts and denominators. Log-transformed RRs were pooled using inverse-variance weighting and DerSimonian-Laird random-effects models. Heterogeneity was assessed with Cochran Q and I^2^. A 95% prediction interval was calculated for the primary outcome to describe the expected range of effects in a new comparable setting. Prespecified subgroup analyses were planned for immune status; however, CD4-specific data were synthesized narratively when comparator definitions, thresholds, or extractable event counts were not sufficiently standardized for a valid pooled estimate.

### Sensitivity and publication-bias analyses

Sensitivity analyses included sequential leave-one-out analyses, a fixed-effect model, and restriction to high-quality studies. Publication bias and small-study effects were assessed by visual inspection of funnel plots and Egger regression for the primary hospitalization analysis because 13 studies were included. Funnel-plot findings were interpreted cautiously because observational studies and heterogeneous hospitalization practices can produce asymmetry unrelated to publication bias.

### Software

Analyses were performed in R and independently reproduced in Python for numerical verification. Figures were regenerated directly from the extracted event counts to ensure consistency between tables and graphical displays.

### Ethics statement

This study used previously published aggregate data and did not involve identifiable individual-level information. Ethical approval and informed consent were not required.

## Results

### Study selection

**FIGURE 1:**
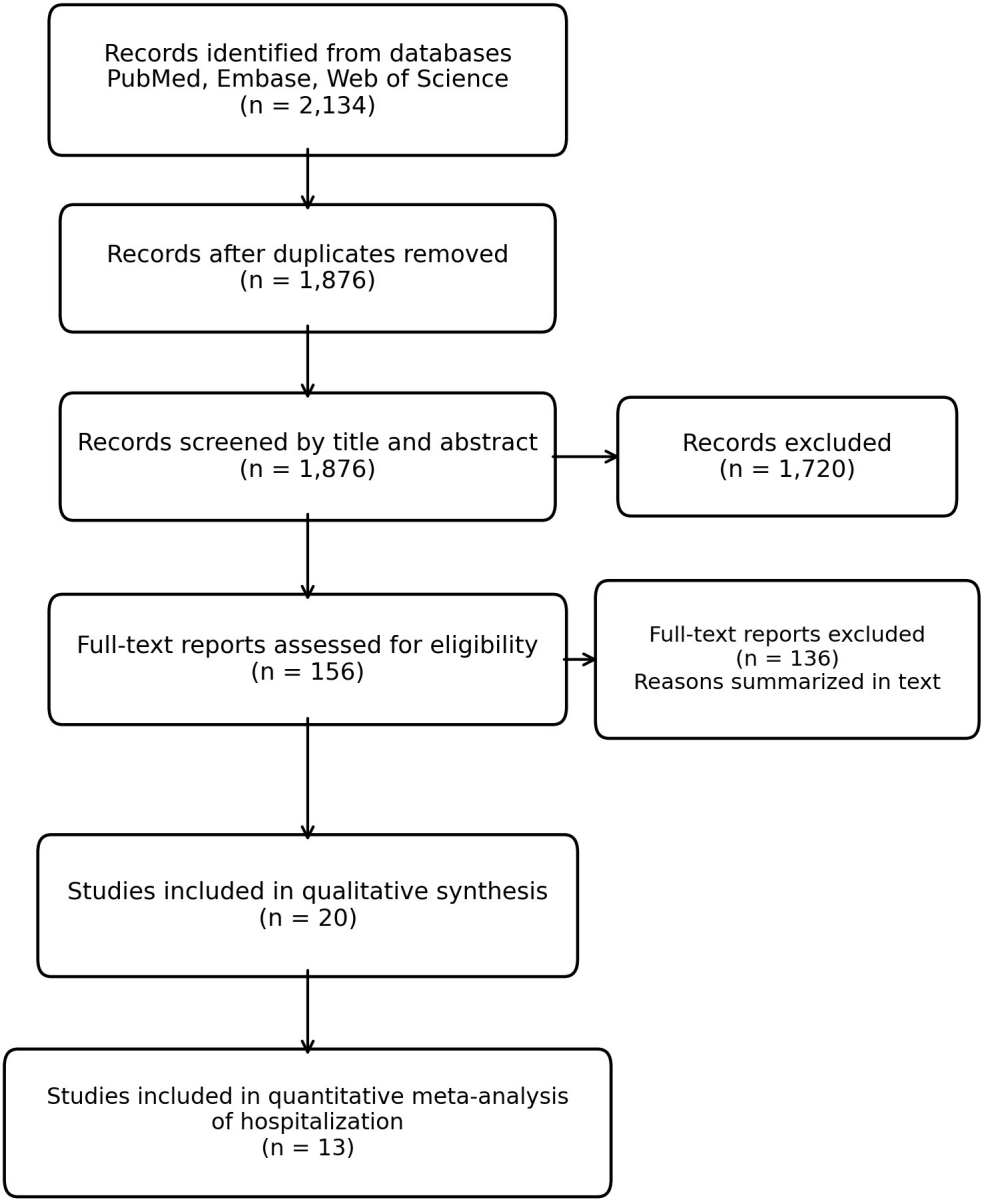
PRISMA flow diagram of study selection. Of 2,134 records identified, 20 studies were included in qualitative synthesis and 13 in the quantitative meta-analysis. Reasons for exclusion of 136 full-text reports are detailed.

The database search identified 2,134 records. After removal of duplicates, 1,876 records were screened by title and abstract; 1,720 were excluded. Of 156 full-text reports assessed for eligibility, 136 were excluded, primarily because they lacked HIV-stratified outcomes, lacked extractable outcome data, used an ineligible design, or represented overlapping populations. Twenty studies were included in the qualitative synthesis and 13 in the quantitative meta-analysis of hospitalization (Figure 1).

### Study characteristics

The 13 studies included in the primary meta-analysis comprised 32,685 participants from North America, Europe, South America, and multinational surveillance systems (Table 1). Most were retrospective cohort or surveillance analyses; cross-sectional and propensity-score matched analyses were also represented. Sample sizes ranged from fewer than 100 to several thousand participants. The proportion of people living with HIV varied substantially, reflecting differences in outbreak patterns, referral setting, sexual-health clinic ascertainment, and regional epidemiology.

**TABLE 1:** Characteristics and extracted hospitalization data for studies included in the primary meta-analysis.

Study	Year	Country/region	Study design	HIV+ *n*	Non-HIV *n*	Hospitalized HIV+ *n*	Hospitalized non-HIV *n*	Reference
Hoffmann	2023	Germany	Retrospective cohort	256	58	7	3	(15)
Angelo	2023	Multinational (GeoSentinel)	Cross-sectional study	92	134	13	17	(3)
Ramirez-Olivencia	2024	Spain	Retrospective cohort	719	659	19	39	(16)
Ramirez-Soto	2024	Peru	National retrospective cohort	2123	1438	154	38	(17)
Krug	2023	France	National surveillance analysis	216	746	6	29	(18)
Garneau	2023	United States	Retrospective cohort	46	54	10	7	(19)
Martin-Iguacel	2023	Spain (Catalonia)	Population-wide retrospective cohort	842	1280	22	22	(5)
Silva	2023	Brazil	Prospective observational cohort	213	196	24	19	(20)
Schildhauer	2023	United States (California)	Retrospective surveillance cohort	1878	2733	140	120	(21)
Henao-Martinez	2023	Multinational	Propensity-score matched analysis	487	990	51	38	(22)
Philpott	2024	United States (Georgia)	Retrospective cohort/surveillance	1124	797	86	37	(6)
Laurenson-Schafer	2023	WHO member states	Global surveillance analysis	6296	8986	369	468	(1)
Chastain	2023	United States	Retrospective cohort	93	229	10	9	(23)

HIV+, people living with human immunodeficiency virus. Non-HIV, individuals without HIV infection. Counts reflect extractable aggregate data used for the hospitalization meta-analysis.

The studies also differed in healthcare setting and case ascertainment. Some datasets were population-wide or surveillance-based, whereas others reflected referral-center or urban cohorts enriched for clinically recognized disease. These differences were expected to influence between-study heterogeneity, especially because hospitalization thresholds vary by healthcare system, outpatient treatment access, clinician concern, and local practice. Risk-of-bias assessment of the included 13 suduies was shown in Table 2.

**TABLE 2:** Risk-of-bias assessment using the Newcastle-Ottawa Scale.

Study	Selection (0–4)	Comparability (0–2)	Outcome (0–3)	Total score
Hoffmann	4	2	3	9
Angelo	3	1	2	6
Ramirez-Olivencia	4	2	3	9
Ramirez-Soto	4	2	3	9
Krug	4	2	3	9
Garneau	3	1	2	6
Martin-Iguacel	4	2	3	9
Silva	4	2	3	9
Schildhauer	4	2	3	9
Henao-Martinez	4	2	3	9
Philpott	4	2	3	9
Laurenson-Schafer	4	2	3	9
Chastain	3	1	2	6

Studies scoring ≥ 7 were considered high quality; scores of 5–6 were considered moderate quality.

### Primary outcome: hospitalization risk by HIV status

**FIGURE 2:**
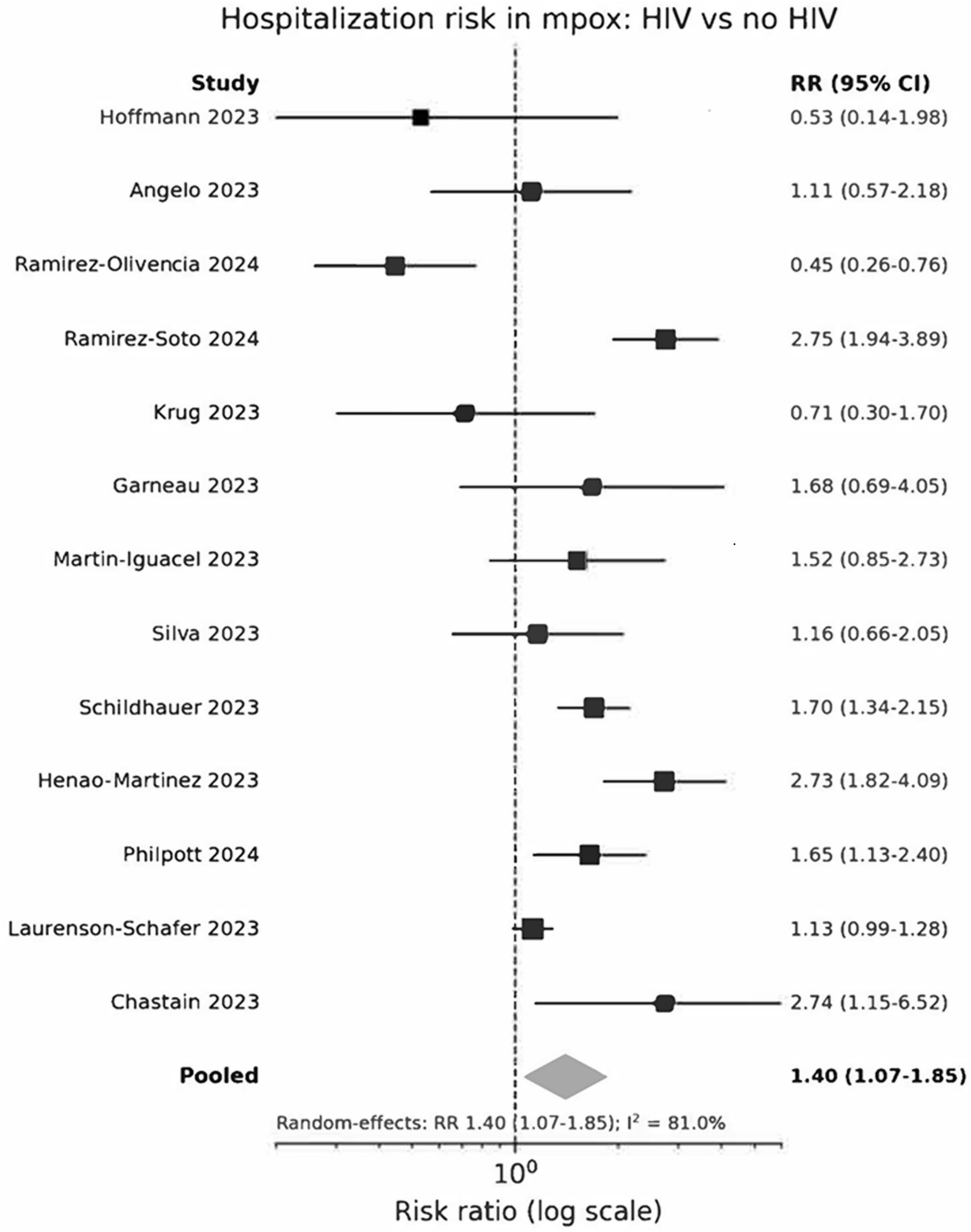
Forest plot of hospitalization risk in mpox by HIV status. Risk ratios (RRs) were pooled using a DerSimonian-Laird random-effects model. HIV infection was associated with increased hospitalization risk (RR 1.40, 95% CI 1.07–1.85; I^2^ = 81.0%).

Across the 13 studies, HIV infection was associated with increased hospitalization risk among individuals with mpox. The random-effects pooled Relative Risk (RR) was 1.40 (95% CI, 1.07–1.85; *p* = 0.015), indicating an approximately 40% higher relative risk of hospitalization among people living with HIV compared with individuals without HIV (Figure 2 and Table 3).

**TABLE 3:** Summary meta-analysis and sensitivity results.

Analysis	Studies	Participants	Effect measure	Pooled estimate	95% CI	95% prediction interval	I^2^ (%)	tau2	*P*-value
Primary random-effects model	13	32685	RR	1.40	1.07–1.85	0.54–3.65	81.0	0.169	0.015
High-quality studies only	10	32037	RR	1.35	0.99–1.84	0.47–3.86	85.0	0.183	0.060
Fixed-effect sensitivity model	13	32685	RR	1.36	1.24–1.49	Not applicable	81.0	Not applicable	0.000

RR, risk ratio; CI, confidence interval. Complete leave-one-out sensitivity data are shown in Supplementary Appendix 1.

Heterogeneity was substantial (I^2^ = 81.0%; Cochran *Q* = 63.15; *p* < 0.001). The 95% prediction interval (0.54–3.65) crossed the null, indicating that the magnitude and direction of the association may vary in future settings depending on case mix, hospitalization practice, vaccination status, HIV disease control, and healthcare access.

### Immune status, CD4 count, and HIV disease control

CD4-stratified data were clinically informative but not sufficiently standardized for a valid pooled CD4-specific meta-analysis. We attempted to pool CD4-stratified hospitalization data using thresholds of <200 cells/mm^3^ and <350 cells/mm^3^, but heterogeneity in threshold definitions, denominator reporting, and outcome presentation precluded valid quantitative synthesis. The reasons were methodological: thresholds varied across studies, some reports used CD4 < 350 cells/mm^3^ rather than <200 cells/mm^3^, denominators were sometimes limited to people living with HIV, and several severe-disease reports were case series or overlapping surveillance populations. Accordingly, the CD4-specific evidence was synthesized narratively rather than presented as a pooled RR.

**FIGURE 3:**
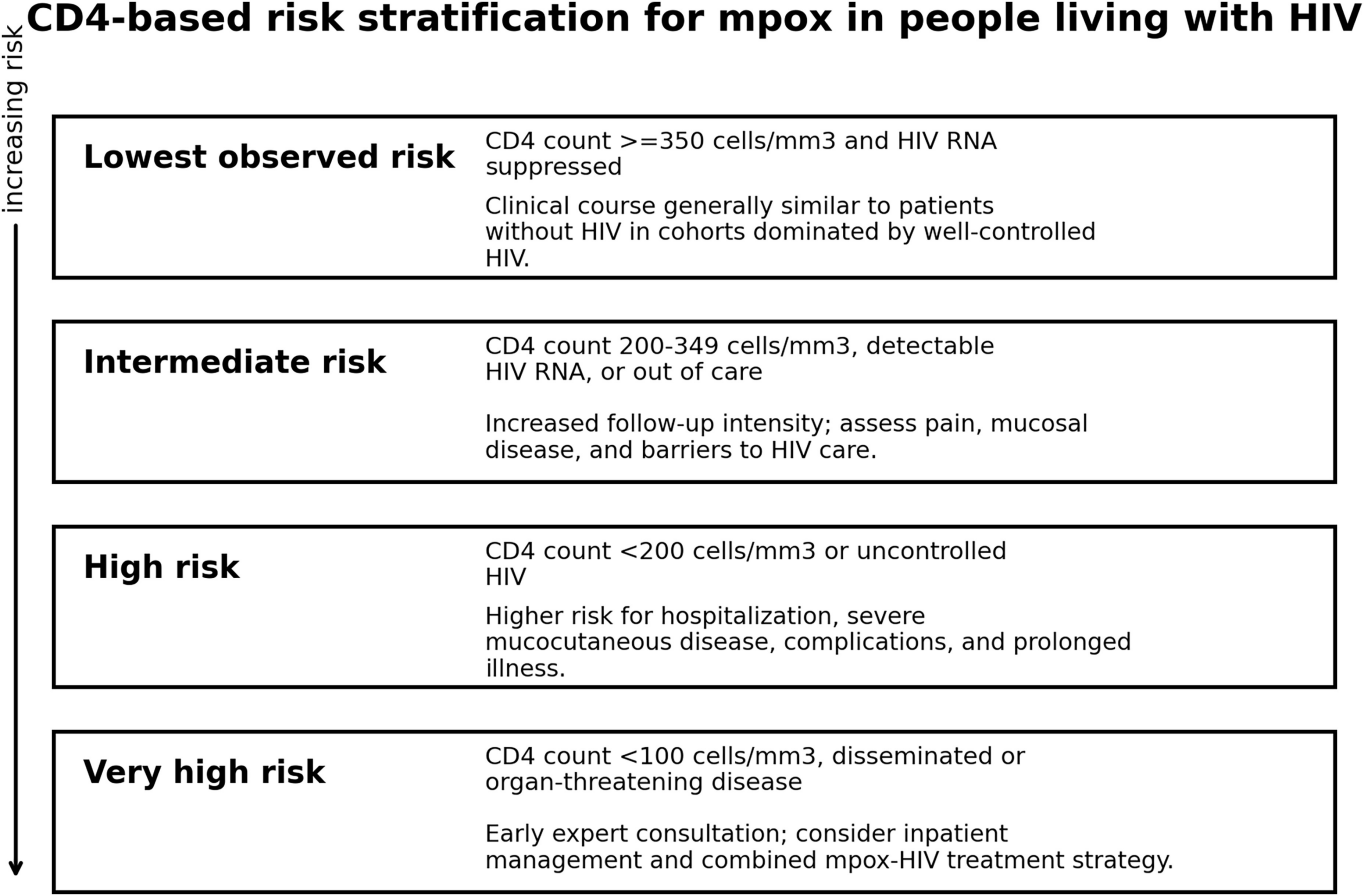
CD4-based risk stratification framework for mpox in people living with HIV. Evidence from included studies is summarized into risk strata based on CD4 count, viral suppression, and engagement in HIV care. This is a qualitative evidence map, not a pooled meta-analysis.

The direction of evidence was consistent. In the Georgia population-level analysis, people living with HIV and CD4 counts below 350 cells/mm^3^ had higher hospitalization risk whether viral load was suppressed or unsuppressed; those not engaged in HIV care were also at increased risk (6). The global advanced-HIV case series showed that severe and fatal mpox clustered in patients with low CD4 counts, particularly below 200 cells/mm^3^ (4). New York City experience further supported a phenotype of prolonged, multiorgan, treatment-refractory disease in people with advanced HIV (7). In contrast, Catalonia and Spain-based cohorts dominated by people with preserved CD4 counts and viral suppression found outcomes similar to those without HIV (5, 13). These findings support CD4-based risk stratification rather than binary HIV classification (Figure 3).

### Sensitivity analyses and publication bias

**FIGURE 4:**
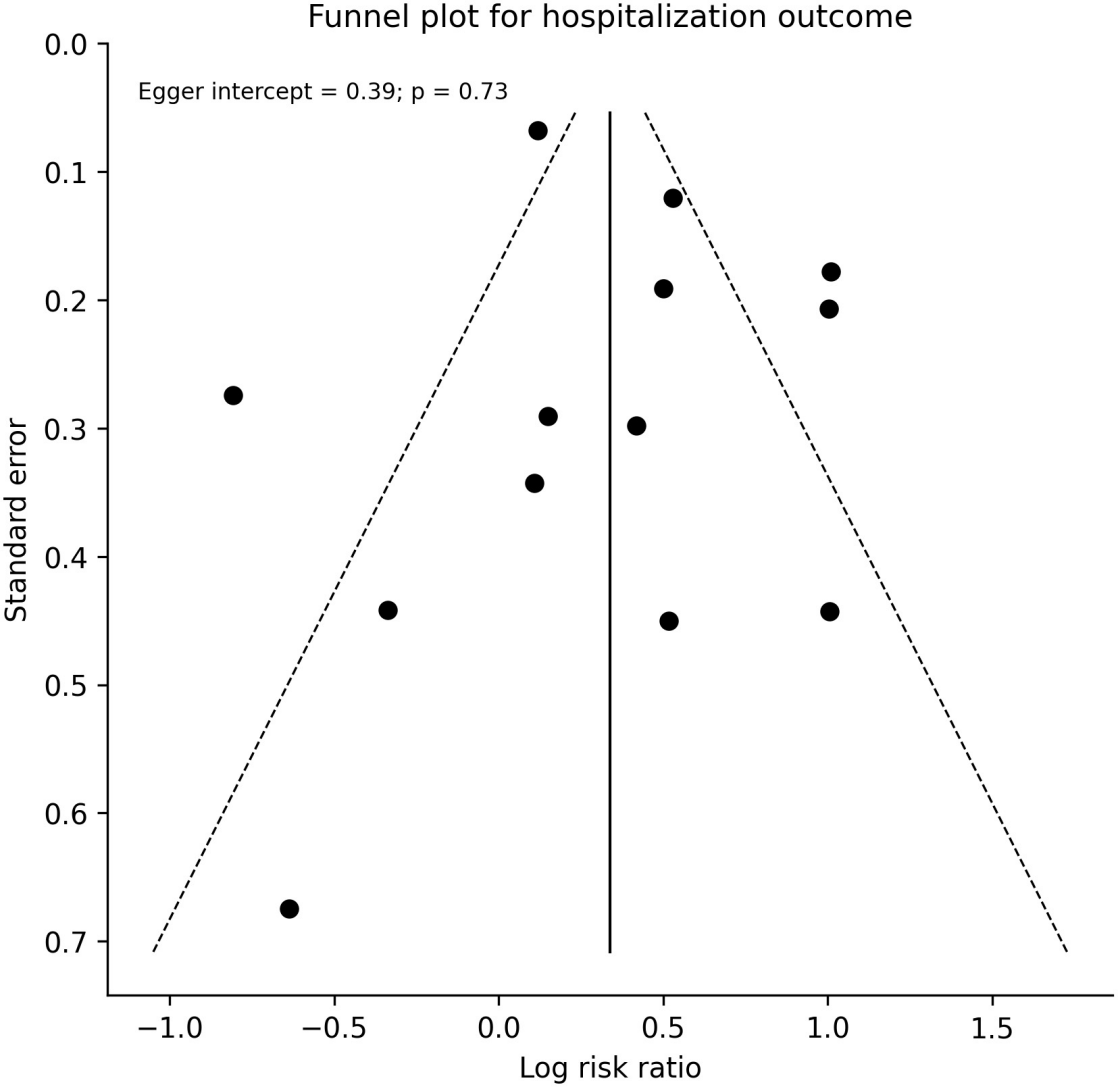
Funnel plot for publication-bias assessment. Each circle represents an included study. Egger’s regression test was non-significant (*P* = 0.73). Cautious interpretation is warranted given observational designs and between-study heterogeneity.

The direction of association was stable in leave-one-out analyses (Supplementary Appendix 1). Restriction to high-quality studies yielded a pooled RR of 1.35 (95% CI, 0.99–1.84). A fixed-effect model yielded an RR of 1.36 (95% CI, 1.24–1.49). Visual inspection of the funnel plot did not suggest marked asymmetry (Figure 4). Egger regression was not significant (intercept, 0.39; *p* = 0.73), but this result should be interpreted cautiously given between-study heterogeneity and observational designs.

## Discussion

In this systematic review and meta-analysis, HIV infection was associated with increased hospitalization risk among patients with mpox. The more important finding, however, is that the excess risk appears to be concentrated in people with immune dysfunction rather than distributing uniformly across all people living with HIV. The primary pooled estimate was statistically significant but heterogeneous, whereas the CD4-informed evidence consistently indicated that low CD4 count, uncontrolled viremia, and disengagement from HIV care identify the subgroup at greatest risk for severe, prolonged, or fatal disease.

This interpretation reconcile apparently discordant findings across the literature. Cohorts dominated by people with well-controlled HIV and preserved CD4 counts often reported similar outcomes between people with and without HIV (5, 13). In contrast, cohorts and case series enriched for advanced HIV showed severe necrotizing skin disease, mucosal disease, secondary infection, multiorgan involvement, prolonged hospitalization, and death (4, 6, 7). HIV serostatus alone is therefore an imprecise exposure: it captures both patients with near-normal immune function and patients with advanced immunosuppression.

The biological plausibility is strong. Orthopoxvirus control depends on intact cellular immunity. Marked CD4 depletion may impair viral clearance, reduce containment of local infection, and increase the risk of dissemination, bacterial superinfection, and persistent viral replication. The clinical pattern reported in advanced HIV resembles a failure of immune containment rather than a uniform consequence of HIV diagnosis. Recent immune-response data further support the concept that viral clearance and inflammatory control differ according to immune status (24).

These results have direct clinical implications. First, assessment of mpox in a person living with HIV should include current or recent CD4 count, HIV RNA, antiretroviral therapy status, and engagement in care. Second, patients with CD4 counts below 200 cells/mm^3^, uncontrolled viremia, or disengagement from HIV care should be considered high risk even before severe manifestations develop. Third, people with preserved immune function and virologically controlled HIV should not automatically be assumed to have severe disease risk equivalent to advanced HIV. This distinction may improve triage, follow-up intensity, and targeted use of limited treatment and public health resources.

The findings align with current guidance (24–27). The NIH opportunistic infection guideline identifies people with HIV who are not virologically suppressed or have CD4 counts below 200 cells/mm^3^ as being at risk for severe mpox and recommends directed antiviral treatment for severe mpox or high-risk patients (25). WHO guidance emphasizes rapid HIV testing, continuation of antiretroviral therapy (ART), and early ART initiation in people with HIV and mpox (28). These recommendations are consistent with the CD4-based framework proposed here.

The manuscript also has public health implications. Mpox services should be integrated with HIV and sexually transmitted infection programs, particularly where delayed HIV diagnosis, low CD4 count, or interruptions in care are common. A syndemic approach allows mpox testing, HIV testing, CD4 and viral-load assessment, linkage or re-linkage to HIV care, vaccination counseling, and treatment triage to occur within the same service pathway. This is more actionable than labeling all people living with HIV as a homogeneous risk category.

This study has several limitations. First, all included studies were observational, and residual confounding is likely. Second, hospitalization is clinically meaningful but imperfect as a severity proxy because admission thresholds differ by healthcare system, outbreak phase, and clinical practice. For example, hospitalization criteria vary between high-income settings such as the United States and middle-income settings such as Brazil, where bed availability and admission practices may influence event capture. Third, CD4 count and HIV RNA were not uniformly reported across studies, limiting quantitative immune-status pooling; moreover, some surveillance datasets may overlap, and careful selection of the most complete dataset was required. Fourth, vaccination status against mpox (e.g., JYNNEOS) was not uniformly reported and could not be adjusted for in the primary meta-analysis. In the few studies that presented vaccination-stratified data, the association between HIV and hospitalization persisted among unvaccinated individuals, but residual confounding or effect modification by vaccination cannot be excluded. Future studies should standardize reporting of vaccination status, CD4 categories, and admission reasons to enable more robust adjustment.

Despite these limitations, the study provides a clinically interpretable synthesis of a heterogeneous literature. The main contribution is not merely the pooled HIV-versus-non-HIV hospitalization estimate, which is broadly consistent with prior meta-analyses (8–10, 29), but the explicit conclusion that CD4 count and HIV disease control are more informative than binary HIV serostatus. Future studies should report standardized CD4 categories, HIV RNA suppression, ART status, vaccination status, tecovirimat exposure, and reasons for hospitalization to permit more precise patient-level or aggregate meta-analysis.

In conclusion, HIV infection is associated with increased hospitalization risk in mpox, but this association is best interpreted through immune status. CD4 count, viral suppression, and engagement in HIV care should be incorporated into mpox risk stratification, clinical management, and public health response. A CD4-based framework avoids overgeneralization, identifies the patients most likely to benefit from intensified management, and aligns mpox control with existing HIV-care infrastructure.

## Data Availability

The original contributions presented in this study are included in the article/Supplementary material, further inquiries can be directed to the corresponding authors.
