# Supplementary material for: Immune dysfunction drives adverse mpox outcomes among people living with human immunodeficiency virus: a systematic review and meta-analysis with CD4-based risk stratification
Source: Front Med (Lausanne). 2026 Jul 29;13:1895518. doi: 10.3389/fmed.2026.1895518 (PMC13463141; doi:10.3389/fmed.2026.1895518)
Supplement: Supplementary file 1 [file Table_1.docx]

Supplementary Appendix 1

Immune dysfunction drives adverse mpox outcomes among people living with human immunodeficiency virus: a systematic review and meta-analysis with CD4-based risk stratification

# Supplementary Methods 1. Search strategies

**PubMed**

((mpox[Title/Abstract] OR monkeypox[Title/Abstract] OR orthopoxvirus[Title/Abstract]) AND (HIV[Title/Abstract] OR "human immunodeficiency virus"[Title/Abstract] OR AIDS[Title/Abstract]) AND (hospitalization[Title/Abstract] OR severity[Title/Abstract] OR severe[Title/Abstract] OR complications[Title/Abstract] OR mortality[Title/Abstract]))

**Embase**

(mpox OR monkeypox OR orthopoxvirus) AND (HIV OR human immunodeficiency virus OR AIDS) AND (hospitalization OR severity OR severe disease OR complications OR mortality)

**Web of Science**

TS=((mpox OR monkeypox OR orthopoxvirus) AND (HIV OR "human immunodeficiency virus" OR AIDS) AND (hospitalization OR severity OR complications OR mortality))

# Supplementary Table S1. Extracted data used in the primary meta-analysis

| **Study** | **Year** | **Country/region** | **Study design** | **HIV+ n** | **Non-HIV n** | **Hospitalized HIV+ n** | **Hospitalized non-HIV n** | **Reference** |
| --- | --- | --- | --- | --- | --- | --- | --- | --- |
| Hoffmann | 2023 | Germany | Retrospective cohort | 256 | 58 | 7 | 3 | 15 |
| Angelo | 2023 | Multinational (GeoSentinel) | Cross-sectional study | 92 | 134 | 13 | 17 | 3 |
| Ramirez-Olivencia | 2024 | Spain | Retrospective cohort | 719 | 659 | 19 | 39 | 16 |
| Ramirez-Soto | 2024 | Peru | National retrospective cohort | 2123 | 1438 | 154 | 38 | 17 |
| Krug | 2023 | France | National surveillance analysis | 216 | 746 | 6 | 29 | 18 |
| Garneau | 2023 | United States | Retrospective cohort | 46 | 54 | 10 | 7 | 19 |
| Martin-Iguacel | 2023 | Spain (Catalonia) | Population-wide retrospective cohort | 842 | 1280 | 22 | 22 | 5 |
| Silva | 2023 | Brazil | Prospective observational cohort | 213 | 196 | 24 | 19 | 20 |
| Schildhauer | 2023 | United States (California) | Retrospective surveillance cohort | 1878 | 2733 | 140 | 120 | 21 |
| Henao-Martinez | 2023 | Multinational | Propensity-score matched analysis | 487 | 990 | 51 | 38 | 22 |
| Philpott | 2024 | United States (Georgia) | Retrospective cohort/surveillance | 1124 | 797 | 86 | 37 | 6 |
| Laurenson-Schafer | 2023 | WHO Member States | Global surveillance analysis | 6296 | 8986 | 369 | 468 | 1 |
| Chastain | 2023 | United States | Retrospective cohort | 93 | 229 | 10 | 9 | 23 |

# Supplementary Table S2. Leave-one-out sensitivity analysis

| **Excluded study** | **RR** | **95% CI lower** | **95% CI upper** | **I2 (%)** | **tau2** | **P value** |
| --- | --- | --- | --- | --- | --- | --- |
| Hoffmann | 1.449 | 1.097 | 1.912 | 82.019 | 0.167 | 0.009 |
| Angelo | 1.425 | 1.067 | 1.904 | 82.485 | 0.177 | 0.016 |
| Ramirez-Olivencia | 1.564 | 1.211 | 2.020 | 76.184 | 0.123 | 0.001 |
| Ramirez-Soto | 1.312 | 1.005 | 1.712 | 76.237 | 0.134 | 0.046 |
| Krug | 1.458 | 1.100 | 1.933 | 81.968 | 0.168 | 0.009 |
| Garneau | 1.388 | 1.043 | 1.847 | 82.519 | 0.175 | 0.025 |
| Martin-Iguacel | 1.391 | 1.039 | 1.863 | 82.540 | 0.180 | 0.027 |
| Silva | 1.423 | 1.062 | 1.906 | 82.498 | 0.180 | 0.018 |
| Schildhauer | 1.363 | 0.993 | 1.871 | 81.389 | 0.215 | 0.056 |
| Henao-Martinez | 1.316 | 1.001 | 1.731 | 78.501 | 0.147 | 0.050 |
| Philpott | 1.375 | 1.018 | 1.858 | 82.275 | 0.190 | 0.038 |
| Laurenson-Schafer | 1.432 | 1.048 | 1.957 | 76.792 | 0.205 | 0.024 |
| Chastain | 1.352 | 1.020 | 1.790 | 81.853 | 0.167 | 0.036 |

# Supplementary Table S3. PRISMA 2020 checklist mapping

| **PRISMA item** | **Requirement** | **Location in manuscript** |
| --- | --- | --- |
| Title | Identify the report as a systematic review/meta-analysis | Title page |
| Abstract | Structured summary | Abstract |
| Rationale and objectives | Explain rationale and objectives | Introduction |
| Eligibility criteria | Specify inclusion/exclusion criteria | Methods |
| Information sources | Specify databases and dates | Methods; Supplementary Methods 1 |
| Search strategy | Present full strategies | Supplementary Methods 1 |
| Selection process | Describe screening process | Methods; Figure 1 |
| Data collection process | Describe extraction process | Methods |
| Data items | List outcomes and variables | Methods |
| Risk of bias | Describe assessment tool | Methods; Table 2 |
| Effect measures | Specify RR and 95% CI | Methods |
| Synthesis methods | Describe random-effects model and heterogeneity | Methods |
| Reporting bias assessment | Describe funnel plot and Egger regression | Methods; Figure 4 |
| Certainty assessment | Not formally performed | Limitations |
| Study selection | Report screening results | Results; Figure 1 |
| Study characteristics | Describe included studies | Results; Table 1 |
| Risk of bias in studies | Present NOS scores | Table 2 |
| Results of syntheses | Present pooled estimates | Results; Table 3 |
| Reporting biases | Present funnel plot/Egger results | Results; Figure 4 |
| Discussion | Interpret findings and limitations | Discussion |
| Registration and protocol | Protocol not registered | Methods |
| Support | Funding statement | Funding |
| Competing interests | Declare conflicts | Conflict of interest |
| Availability of data | Describe data availability | Data availability statement |

# Supplementary reproducibility note

The primary hospitalization analysis used the event counts and denominators shown in Supplementary Table S1. Risk ratios were log-transformed, pooled with inverse-variance random-effects weighting, and back-transformed for display. Prediction intervals and leave-one-out analyses were calculated from the same extracted dataset.
